# Zookeepers’ Perception of Zoo Canid Welfare and its Effect on Job Satisfaction, Worldwide

**DOI:** 10.3390/ani10050916

**Published:** 2020-05-25

**Authors:** Giacomo Riggio, Federica Pirrone, Elia Lunghini, Angelo Gazzano, Chiara Mariti

**Affiliations:** 1 Vethos–Veterinary Clinical Ethology, 00182 Rome, Italy; giacomo.riggio@phd.unipi.it; 2 Department of Veterinary Sciences, University of Pisa, 56124 Pisa, Italy; angelo.gazzano@unipi.it; 3 Department of Veterinary Medicine, University of Milan, 20133 Milan, Italy; federica.pirrone@unimi.it; 4 A.I.G.ZOO–Italian Association of Zookepers, 51100 Pistoia, Italy; presidenza@aigzoo.it

**Keywords:** zookeepers, animal welfare, canids, gender, five freedoms, bond, job satisfaction

## Abstract

**Simple Summary:**

An important part of zookeepers’ work consists of monitoring and assessing zoo animal welfare. In this study, we investigated zookeepers’ perception of the importance and fulfilment of zoo canids’ fundamental freedoms, and how it correlates with their job satisfaction. Our findings suggest that zookeepers perceive zoo canids’ freedoms to be important, but not as much guaranteed, especially those related to psychological aspects of welfare. However, zookeepers’ female gender is associated with a more positive perception of both the importance and fulfilment of these freedoms. Overall, zookeepers who reported a more positive perception of the fulfilment of zoo canids’ freedoms, as well as a stronger keeper–animal bond, appeared to be more satisfied with their job, in general. Our findings suggest that zoos should put more efforts in ensuring their canids the protection of fundamental freedoms, especially those related to psychological aspects of welfare. Furthermore, enhancing zoo canid welfare, as well as promoting management procedures that allow the development of proper keeper–canid interactions, may increase job satisfaction among zookeepers.

**Abstract:**

Recently, zookeepers’ role in monitoring and assessing zoo animal welfare is gaining importance. One hundred-sixteen zoo canid keepers responded to an online questionnaire aimed at assessing, on a 1 to 5 scoring scale, their perception of the importance and fulfilment of the Brambell’s Freedoms for zoo canids, the bond with canids under their care, and their level of job satisfaction. Results showed that zookeepers perceive the Brambell’s Freedoms as highly important (median = 5, min–max = 3–5), but not equally guaranteed (median = 3, min–max = 1–5, *p* < 0.01). Although there was no difference in their perception of the importance of each freedom, those related to psychological issues (median = 3, min–max = 1–5) were perceived as significantly less guaranteed than those addressing physical needs (median = 4.5, min–max = 1–5, Mann–Whitney U test, *p* < 0.01). Female zookeepers tended to perceive all freedoms as more important (Ordinal Logistic Regression model, *p* = 0.009), as well as more guaranteed (Ordinal Logistic Regression model, *p* = 0.007), than male zookeepers. Regardless of gender, a more positive perception of the Brambell’s Freedoms for zoo canids was associated with higher job satisfaction (Mann–Whitney U test, *p* < 0.01, ρ = 0.241). The latter was also positively correlated with zookeepers’ perception of the strength of the bond with the canids under their care (Spearman Rho’s correlation, *p* = 0.01, ρ = 0.230). Our results highlight the need for zoos to focus on guaranteeing psychological welfare of their canids. Enhancing animal welfare may increase zookeepers’ job satisfaction.

## 1. Introduction

Public awareness about animal welfare has been progressively growing over the last few decades [1]. In response, international and national associations of zoos and aquaria have adopted internal policies aimed at achieving and maintaining high welfare standards for the animals under their care [2]. For several years, zoo animal welfare assessment has followed a resource-based approach, which focused on the evaluation of environmental and management factors that may indirectly compromise welfare [3]. Although a resource-based approach helps address and eventually minimize those environmental factors that may jeopardise welfare at the species level, it does not take into account individual needs, preferences and affective states [2]. More recently, zoo facilities and welfare researchers have progressively switched their focused towards animal-based types of assessment in order to be able to ensure individual well-being [2,4,5,6]. In this scientific context, zookeepers have become a valuable tool for assessing zoo animals’ level of welfare [2,7,8]. By the nature of their job, they have the opportunity to spend large amounts of time observing the animals under their care and gain valuable information that can be used to address welfare issues and identify strategies for improvement [2,3,7,8]. For such reason, zookeeper’s role as proxy evaluators in zoo animal studies that aimed to assess the animal’s temperament, preference, behaviour and well-being, has become progressively more common over the years [2,3,9,10,11,12,13,14,15,16,17,18,19]. Furthermore, major zoos and aquaria organizations recommend their members to use zookeepers in systematic internal assessments of their animals’ level of welfare. For instance, the Association of Zoos and Aquaria (AZA) accredited facilities are required to implement Institutional Animal Welfare Processes (IAWP) by using their staff members to monitor and report welfare risk factors [3,20]. Similarly, the World Association of Zoos and Aquaria (WAZA) promotes the implementation of internal welfare assessments performed by experienced staff members [21]. However, this type of assessments are subjective and may be affected by the observer’s personal attitude and perception towards animal welfare [17,22,23]. As shown in previous studies, several factors may affect people’s attitude towards animals, as well as the perception of their welfare. For instance, female gender is usually associated with higher concern of animal welfare issues in both the general public [24,25,26,27,28,29] and those professional categories that may be directly involved in the assessment of animal welfare [22,30,31,32,33,34,35]. Similarly, age [24,32], geographical origin [36], socioenvironmental context [37], educational level and background [22,35,38], religion [24], personality [39,40], companion animal ownership [24,25,41,42,43,44], familiarity/experience with animals [25,30,36,45] and strength of the human–animal bond [25,46] have been found to affect people’s attitude towards animals. Furthermore, attitude towards animals may vary in relation to the species considered [24,47], to its perceived ecological value (i.e., more charismatic vs. less charismatic) [47,48] and to the purpose of its use (i.e., farm vs. companion vs. laboratory vs. zoo) [30,48].

Job satisfaction relates to the pleasure and gratification experienced within the working environment, and of course, it can be affected by a variety of financial, social and environmental factors [49]. In animal care professionals, job satisfaction may also be affected by the quality of the relationship with the animals under their care and by their perception of the animals’ well-being [50]. Zookeepers have also reported to experience emotional connection with their animal counterparts [51,52]. Nonetheless, to date, only a few studies have focused on assessing the impact of human–animal relationship and the level of animal welfare on zookeepers’ job satisfaction [53,54,55].

The aim of this study was twofold. First, we assessed zookeepers’ perception of zoo canid welfare, in relation to background and demographic factors. Second, we investigated whether zookeepers’ perception of zoo canid welfare and keeper–canid relationship may have an impact on their job satisfaction.

## 2. Materials and Methods 

### 2.1. Data Collection

Data were collected from June 2018 to June 2019 with the help of national and international zookeeper associations as well as online zookeeper forums that shared the questionnaire among their members. The questionnaire was compiled online, anonymously, after having provided consent to participation, by zookeepers who had experience in taking care of canids. The original questionnaire comprised 86 questions divided into 6 major sections.

Except for the demographic section, all the others included only 1 to 5 Likert-type scale questions. Cronbach’s alpha was used to estimate internal consistency and only questions from three subscales, among those with an acceptable score (Cronbach’s alpha > 0.70), were retained and further analysed for the purpose for this study (Supplementary Text S1).

The first and second subscales aimed at assessing, respectively, zookeepers’ perception of the importance and fulfilment of each of the Brambell’s Five Freedoms [56], for zoo canids (e.g., In your opinion, how important are the freedoms listed in the Brambell Report for the welfare of captive wild canids, in zoos?). For this purpose, a modified version of Mariti et al.’s [30] questionnaire aimed to assess veterinary students’ perception of pets and farm animals’ welfare was used. The third subscale comprised questions about animal welfare specifically in the zoo where the respondents were employed.

### 2.2. Respondent’s Demographics

The questionnaire was completed by 116 zoo canid keepers; 86.0% of them were still working with zoo canids at the time they filled out the questionnaire, the rest had worked with zoo canids during the last 5 years. Respondents were from 16 different countries across 4 continents (Europe, America, Asia and Oceania). Among all nationalities, the most represented respondents were from the United States (25.0%), the United Kingdom (17.2%), Italy (14.7%), Australia (12.1%), The Netherlands (8.6%) and Germany (4.3%). Their age ranged from 18 to 54 years (mean 31.86 ± SE 0.71 years) with the majority of them being women (72.4%). Years working as a zookeeper ranged from approximately 1 to 30 (median = 6.5 years), whereas time working specifically as a zoo canid keeper ranged from 6 months to 24 years (median = 4.25 years). Almost half of them (48.3%) had worked only in one zoo facility, 25.0% in two, 17.2% in three, 6.9% in four, 1.7% in five and 0.9% in six. As for their education level, more than a half (56%) had a university degree, 14.7% attended postgraduate studies, 20.7% had a high-school degree and 8.6% had none of the previous. The vast majority of them (85.3%) attended at least one course on animal welfare related topics. Among the reasons listed for becoming a zookeeper the most frequent was “Love for animals” (87.1%), followed by “Interest in species conservation” (56.9%). Only 3.5% of respondents did not include either of them and became zookeepers because it was the “Only job found” or because “They were introduced by an acquaintance who already worked in the sector.” For 68.1% of them, working with canids rather than other animals was not a specific choice. More than a half (56.1%) of the keepers took care of more than one species. The most represented species was the wolf (54.3%), followed by the fox (40.3%) and the African wild dog (30.1%). Time spent in proximity (visual contact) of the animals varied from 0 to 420 min (median = 60). Most of the respondents (84.5%) owned or had owned a dog as a pet.

### 2.3. Statistical Analysis

Statistical analysis was conducted using SPSS version 25.0 (IBM Corp., Armonk, NY, USA). Values are reported as medians. The total scores were calculated, and Cronbach’s alpha was used to estimate internal consistency [57]. For ease of interpretation, the scores were converted to the percentage of maximum possible (POMP) [58], where 0 and 100 represent the lowest and highest possible scale scores, respectively.

Using nonparametric statistics (Mann–Whitney and Kruskal–Wallis), we tested for differences in scoring the tendency of keepers as a preliminary screening. Spearman Rho’s correlation was computed to determine associations between variables. Bonferroni correction was made adjusting the *p*-value by multiplying *p* × (# comparisons).

Ordinal Logistic Regression (OLR) models were then run to assess the association between each scale score (dependent variable, Y) and each of the potential risk factors (independent variables, X), for which, there were significant differences in the answering tendency of keepers. Model Fitting was tested with the −2 log likelihood (−2LL) method for the intercept-only and final models. The Wald χ 2 test was performed to detect statistically significant predictors. The odds ratio (OR) for the predictors was calculated to assess the strength of these relationships. The Test of Parallel Lines was used to evaluate the proportional odds assumption. A two-sided *p* < 0.050 was deemed to be statistically significant.

## 3. Results

### 3.1. Demographics and Importance of Brambell’s Five Freedoms

Zookeepers’ median POMP score on the importance of the Brambell’s Five Freedoms was 100 out of 100 (min 40–max 100), with a good Cronbach’s alpha score of 0.81. Among all the demographic factors investigated (i.e., gender, age, country of work, primary motivation for working as a zookeeper, years working as a zookeeper, years working as a canid keeper, educational level, attendance to animal welfare courses, number of zoos worked at, species of canids taken care of, dog ownership and time spent in proximity with zoo canids), statistically significant differences were found in relation to gender (Mann–Whitney U = 918000, *p* = 0.004) and to the country where the respondents worked (Kruskal–Wallis test χ^2^ = 26.569, df = 16, *p* < 0.050). 

The variables for which there were differences in the scoring tendencies of keepers were submitted to the OLR analysis. The explanatory variables improved the model, because unexplained variation decreased from 364.31 in the model with only the intercept to 311.01, and the difference (53.30) was statistically significant (*p* < 0.019). There was strong evidence of an association between zookeepers’ scores and gender (Table 1).

The odds for female zookeepers to score high on the subscale related to the importance of the Brambell’s Five Freedoms were about 4.82 greater than the odds for males (Table 1). The proportional odds assumption held because the significance of Chi-Square statistic in the Test for Parallel Lines was > 0.05 (−2 log likelihood = 71.596, χ^2^ = 239.41, df = 306, *p* = 0.990).

### 3.2. Demographics and Fulfilment of Brambell’s Five Freedoms

Zookeepers’ median POMP score on the fulfilment of the Brambell’s Five Freedoms was 70 out of 100 (min 10–max 100), with a good Cronbach’s alpha score of 0.82. Demographic factors, such as gender, age, country of work, primary motivation for working as a zookeeper, years working as a zookeeper, years working as a canid keeper, educational level, attendance to animal welfare courses, number of zoos worked at, species of canids taken care of, dog ownership and time spent in proximity with zoo canids were investigated. As for the subscale related to the importance, statistically significant differences were found in relation to gender (Mann–Whitney U = 862000, *p* = 0.003) and to the country where the respondents worked (Kruskal–Wallis test χ^2^ = 36.760, df = 16, *p* = 0.002). Both variables were submitted to the OLR analysis. The likelihood ratio test revealed an improvement over the intercept-only model, demonstrating that the logistic model provided a better fit to the data (−2 log likelihood of the intercept-only model = 579.93; −2 log likelihood of the final model = 481.40, χ^2^ = 98.53, df = 34, *p* = 0.001). Only gender was a predictor of the zookeepers’ scores. In this case, the odds for female zookeepers to score high on the subscale related to the fulfilment of the Brambell’s Five Freedoms were about 3.97 greater than those for males (Table 2). The −2LL parallel regression assumption yielded χ^2^ statistics > 0.05, indicating that the proportional odds assumptions for the full-model was upheld (−2 log likelihood = 0.001, χ^2^ = 481.40, df = 442, *p* = 0.095).

### 3.3. Correlations between Importance and Fulfilment of the Brambell’s Five Freedoms

A positive correlation between the scores on the importance and the fulfilment of Brambell’s Five Freedoms was found (*p* = 0.005, ρ = 0.333). The median POMP value for the subscale related to the importance was significantly higher than that related to the fulfilment (*p* < 0.001). Furthermore, although zookeepers perceived all freedoms to be equally important, they perceived some freedoms to be less guaranteed than others (*p* < 0.001). Specifically, Freedom to have an adequate physical environment was perceived significantly less guaranteed than Freedom from hunger and thirst (Mann–Whitney U = 2.060, *p* < 0.001) and Freedom from pain, injury and disease (Mann–Whitney U = -.121, *p* < 0.001). Freedom from fear and distress was perceived significantly less guaranteed than Freedom from hunger and thirst (Mann–Whitney U = 1.793, *p* < 0.001) and Freedom from pain, injury and disease (Mann–Whitney U = 0.853, *p* < 0.001). Freedom to express normal behaviour for the species was perceived as significantly less guaranteed than Freedom from hunger and thirst (Mann–Whitney U = 1.823, *p* < 0.001) and Freedom from pain, injury and disease (Mann–Whitney U = 0.884, *p* < 0.001). Lastly, Freedom from injury and disease was perceived as less guaranteed than Freedom from hunger and thirst (Mann–Whitney U = 0.940, *p* < 0.001).

### 3.4. Zookeepers’ Satisfaction with Zoo Animal Welfare and with Their Job

Respondents were generally satisfied with the level of animal welfare in the zoo where they worked (median = 4, min–max = 1–5) and with their job, in general (median = 4, min–max = 1–5). Zookeepers’ satisfaction with the level of welfare of the animals in the zoo where they worked was positively correlated with their perception of the fulfilment of the Brambell’s Freedoms (corrected *p* = 0.002, ρ = 0.394). Zookeepers’ satisfaction with their job was positively correlated with the perceived level of fulfilment of the Brambell’s Five Freedoms (corrected *p* = 0.02, ρ = 0.241) for zoo canids, with their satisfaction with the level of welfare of the animals in the zoo where they worked (corrected *p* = 0.02, ρ = 0.683) and with the strength of the bond with the canids under their care (corrected *p* = 0.02, ρ = 0.230).

## 4. Discussion

In the last few years, zookeepers have been increasingly involved in the assessment of zoo animal welfare, both in research studies and in welfare monitoring institutional protocols [3,20,21]. For this reason, a better knowledge of their perception of animal welfare in zoos may provide information on how to improve their work.

The results from our study show that there is a significant discrepancy between their perception of the importance and level of protection of the Brambell’s Five Freedoms for zoo canids. In particular, while all freedoms were perceived as highly important, those related to psychological aspects of welfare were perceived as significantly less guaranteed than those related to physical needs. There are two possible explanations to this result, which do not necessarily exclude each other. On one hand, zoos may put more efforts in ensuring good physical rather than psychological health [59,60]. Historically, captive animal welfare assessments have focused on the evaluation of physical indicators, such as body condition [61], pain [62], injuries [63], diseases [52], reproductive success [64,65], mortality [66] and longevity [65], rather than psychological indicators of welfare [59,60]. This is likely because, compared to altered psychological and emotional states, deviations from optimal physical health can be measured through more tangible metrics [59,60]. On the other hand, psychological welfare may be not only harder to asses but also harder to achieve in captive animals. Confinement per se may be a major risk factor for zoo animal welfare, especially for wide-ranging species [67,68], such as several wild canids, for which measures such as environmental enrichment seem to partially improve the level of welfare [69]. Although confinement may only marginally affect physical health, which is usually ensured by systematic assessment of physical conditions and proper veterinary care, it may have a much stronger impact on the animals’ emotional and psychological well-being [67,70]. Despite of the fact that big steps have been taken towards the acknowledgment and the inclusion of psychological and emotional states in animal welfare assessment and management, this finding suggests that psychological aspects of zoo canid welfare may require even more careful consideration than they are currently given. Indeed, the design of this study does not allow to objectively assess the actual fulfilment of the Brambell’s Freedoms, but only to measure how much the respondents perceive such freedoms to be guaranteed for zoo canids. However, as reported in previous studies [2,3,7,71], zookeepers have a unique baggage of experience with zoo animals that allows them to gain in-depth knowledge of their behaviour, needs, temperament and well-being. Therefore, although subjective, zookeepers’ perception of the Brambell’s Freedoms may be regarded, by zoo institutions, as a supportive tool to readily identify aspects of canid welfare that may require improvement. 

The same scoring difference between perceived importance and fulfilment of the Brambell’s Freedoms was observed in both female and male zookeepers’ responses. However, females scored higher than males in both cases. Females’ more positive perception of the importance of the Brambell’s Freedoms is not surprising, as it reflects the findings of several previous studies that identify female gender as a strong predictor of greater concern for animal welfare issues and a more positive attitude towards animals, in general [24,27,28,29,45]. On the contrary, the finding that female zookeepers perceived Brambell’s Freedoms to be also more guaranteed, may appear contradictory. We would have expected female zookeepers to be more demanding in the assessment of animal welfare and, as a consequence, more severe in their judgement on perceived levels of welfare. On one hand, this result may simply reflect their greater commitment and efficacy to guaranteeing their canids’ welfare. This may not be surprising given that women tend to be more sensitive to animal protection [72] and, as a consequence, tend to behave in a way that fulfils the animals’ needs when they are involved in animal caretaking activities [73]. On the other hand, when using animal caretakers’ ratings, the possibility of “self-protection” (i.e., the desire and preference for minimizing the negativity of self-views) and “self-enhancement” (i.e., desire and preference for maximizing the positivity of self-views) biases [74] should always be taken into account [17], especially when assessing aspects that are considered highly important by the respondents, as in the case of female zookeepers of this study, and/or for which the respondents may feel responsible [17].

On the contrary, gender did not affect the respondents’ satisfaction with the level of welfare of the animals under their care. This finding suggests that zookeepers’ satisfaction with animal welfare is related not only to their perception of fulfilment of the Brambell’s Freedoms but also to the delta between the latter and their perceived importance. Accordingly, satisfaction with animal welfare resulted the same between male zookeepers, who perceived the Brambell’s Freedoms as less guaranteed, but also less important, and female zookeepers, who perceived them to be more important, but also more guaranteed.

In the present study, we also investigated the correlation between zookeepers’ perception of zoo canid welfare and their job satisfaction, which can be defined as a measure of the worker’s affective state in relation to his/her job condition, and as such, may act as an indicator of the worker’s well-being [54]. The results suggest that zookeepers’ job satisfaction was positively correlated with a more positive perception of the fulfilment of the Brambell’s Freedoms as well as with a higher satisfaction about the level of welfare of the animals under their care. However, only the latter correlation appeared to be strong. The different strength of correlation seems to suggest the path of results: although the question on the fulfilment of the Brambell’s Freedoms referred to zoo canids in general, the question on satisfaction on the animals’ level of welfare referred specifically to the individuals housed in the zoo where the respondents worked, and only the second more likely has a strong impact on their job satisfaction. Previous studies have suggested that job satisfaction in animal care professionals may be affected by their perception of the affective state of the animals under their care. For instance, perceived happiness and absence of stress in laboratory animals was found to play an important role in their caretakers’ job satisfaction [75]. Similarly, shelter workers’ job-related sadness and frustration were found to be associated with dogs’ poor physical or psychological welfare [50]. As for zookeepers, a study by Carlstead et al. [54] found that their job satisfaction was negatively correlated with the animals’ fear of people. Although several other factors (i.e., financial, social, environmental, etc.) are likely to be involved, our results support the conclusion of previous studies [75] that enhancing animal welfare may increase animal caretakers’ job satisfaction, and consequently, their work-related well-being. Nevertheless, the opposite may also be possible. In fact, as suggested by previous authors, job satisfaction may in turn predispose animal care professionals to more positive emotions and attitudes towards their animals [54] and boost their motivation to provide them with optimal care [75]. Although the presence of a relationship between zoo animal welfare and zookeepers’ work-related well-being seems certain, further research is needed in order to fully comprehend the dynamics through which these factors influence each other.

According to the definition provided by the American Veterinary Medicine Association [76], the human–animal bond is “a mutually beneficial and dynamic relationship between people and animals that is influenced by behaviours that are essential to the health and wellbeing of both.”

While there is extensive scientific evidence that human–animal bonds can be formed between pets and their owners [77], it is still not clear whether the same type of mutually beneficial relationship can occur between captive animals and their caretakers [55]. Nonetheless, the great majority of zookeepers report to experience affective bonds with the animals under their care [51] or describe special emotional connections with specific individuals [52]. Therefore, regardless of the type and depth of the bond, zookeepers seem to experience some kind of emotional benefit from the relationship with the animals they take care of [52]. In accordance with the findings of a recent study on keeper–elephant relationship [53], our results seem to suggest that zookeepers who experience a stronger bond with the canids under their care are more satisfied with their job, in general. A strong bond is also likely to lead the keeper to take better care of animals in the zoo. Therefore, implementing management procedures that allow systematic positive interactions between zookeepers and zoo animals may have a beneficial effect for the well-being of both parties.

This study has some limitations that need to be addressed. As previously mentioned, this study was designed to assess zookeepers’ subjective perception, not to obtain objective data on zoo canid welfare. Although zookeepers’ surveys seem to be a reliable and valid tool to assess various aspects related to zoo animal well-being [2,3,7,8,19], to the best of our knowledge, the Brambell’s Five Freedoms have never been used as a framework to assess zookeepers’ perception of canid welfare. Therefore, objective data that support or confute the results of this study need to be investigated in the future. Moreover, we did not assess how familiar zookeepers were with the Brambell’s Five Freedoms. Nonetheless, the Brambell’s Five Freedoms are a simple welfare framework [78] that is comprehensible even to a nonspecialist public [79]. Besides, 85.3% of our respondents attended at least one welfare course during their career, which makes them likely to have at least basic notion of this widely used welfare framework.

Another limitation of this study is that it focuses on the Canidae family, exclusively. It would be interesting to know whether zookeepers have a similar perception of the importance and fulfilment of the Brambell’s Five Freedoms in relation to different species. Lastly, in order to obtain a greater number of responses, the questionnaire was distributed online. However, this prevented us from knowing the number of zookeepers that received the questionnaire but declined to participate in the study. Furthermore, we could not find data on the number of zoo canid keepers operating across the world neither in scientific literature nor by consulting zookeeper associations. As a consequence, we do not know how representative of the entire zoo canid keeper population our respondents were.

## 5. Conclusions

Results of this study suggest that zookeepers perceived all the Brambell’s Freedoms to be highly important, but not equally guaranteed. In particular, Freedoms related to psychological aspects of welfare were perceived as significantly less guaranteed than those related to physical needs, suggesting that the first ones may require even more careful consideration. An expected gender effect was found, with female zookeepers perceiving the Brambell’s Five Freedoms to be both more important and more guaranteed. Finally, a more positive perception of the fulfilment of the Brambell’s Freedoms, a higher satisfaction with the animals’ level of welfare and a stronger keeper–canid bond, was all associated with greater job satisfaction. Thus, suggesting that the implementation of systematic positive interactions between zookeepers and zoo animals may have a beneficial effect for the well-being of both parties. 

## Figures and Tables

**Table 1:** Ordinal Logistic Regression model result predicting zookeepers’ scores on the Brambell’s Five Freedoms importance scale.

Parameter	B	SE	Hypothesis Test			OR	95% Wald CI for OR	
			Wald Chi-Square	Df	Sig.		Lower	Upper
[Gender = female]	1.573	0.600	6.895	1	0.009	4.821	1.490	15.595
[Gender = male]	0 ^a^					1		

Significance: *p* < 0.05. B: regression coefficient. SE: standard error of the mean, OR: odds ratio, CI: confidence interval. ^a^: This parameter was set to zero because it is redundant.

**Table 2:** Ordinal Logistic Regression model result predicting zookeepers’ scores on the Brambell’s Five Freedoms fulfilment scale.

Parameter	B	SE	Hypothesis Test			OR	95% Wald CI for OR	
			Wald Chi-Square	df	Sig.		Lower	Upper
[Gender = female]	1.380	0.512	7.247	1	0.007	3.974	1.455	10.851
[Gender = male]	0^a^					1		

Significance: *p* < 0.05. B: regression coefficient. SE: standard error of the mean, OR: odds ratio, CI: confidence interval. a: This parameter was set to zero because it is redundant.

## Supplementary Materials

The following are available online at https://www.mdpi.com/2076-2615/10/5/916/s1, Text S1: Questions analysed for the purpose of this study and corresponding scoring system.
