# Supplementary material for: Zookeepers’ Perception of Zoo Canid Welfare and its Effect on Job Satisfaction, Worldwide
Source: Animals (Basel). 2020 May 25;10(5):916. doi: 10.3390/ani10050916 (PMC7278387; doi:10.3390/ani10050916)
Supplement: Supplementary file 1 [file animals-10-00916-s001.pdf]

1. Age

2. Gender

Mark only one oval.

- Male
- Female

3. In which country do you work?

4. How many years have you been working as a zookeeper?

5. What is your education level?

Mark only one oval.

- High school
- University
- Post-graduate studies
- None of them

6. Have you ever attended courses on animal welfare or welfare-related topics?

Mark only one oval.

- Yes
- No

7. How long have you worked/been working with captive canid species?

____

8. To date, do you still work as a canid keeper?

Mark only one oval.

- Yes
- No

9. Did you specifically choose to work with wild canid species?

Mark only one oval.

- Yes
- No

10. How many zoos have you worked in?

____

11. Why did you first choose to work as a keeper?

Tick all that apply.

- [ ] Love for animals
- [ ] Money
- [ ] Interest in species conservation
- [ ] Favourable working shifts
- [ ] Introduced by an acquaintance who already worked in the sector
- [ ] Only job found

Other: [ ] ______________________

12. Have you ever owned a dog?

Mark only one oval.

( ) Yes

( ) No

13. Which group of canids do you take care of?

Tick all that apply.

- [ ] African wild dog
- [ ] Coyote
- [ ] Fox
- [ ] Jackal
- [ ] Wolf
- [ ] Cuon
- [ ] Racoon dog
- [ ] Bush dog
- [ ] Maned wolf

Other: [ ] ______________________

14. On average, how much time (minutes) per day do you spend around (visual contact) the canids you take care of?

____

15. How strong is the average bond you have with zoo canids you take care of?

Mark only one oval.

	1	2	3	4	5	
Very trivial	◯	◯	◯	◯	◯	Very strong

16. Are you satisfied by your job, in general?

Mark only one oval.

	1	2	3	4	5	
Not satisfied at all	◯	◯	◯	◯	◯	Totally satisfied

In your opinion, how important are the freedoms listed in the Brambell Report for the welfare of captive wild canids, in zoos?

17. Freedom from hunger and thirst

Mark only one oval.

	1	2	3	4	5	
Not important at all	◯	◯	◯	◯	◯	Very important

18. Freedom to have an adequate physical environment

Mark only one oval.

	1	2	3	4	5	
Not important at all	◯	◯	◯	◯	◯	Very important

19. Freedom from pain, injury and disease

Mark only one oval.

	1	2	3	4	5	
Not important at all	◯	◯	◯	◯	◯	Very important

20. Freedom to express normal behaviours (for the species)

Mark only one oval.

	1	2	3	4	5	
Not important at all	◯	◯	◯	◯	◯	Very important

21. Freedom from fear and distress

Mark only one oval.

	1	2	3	4	5	
Not important at all	◯	◯	◯	◯	◯	Very important

In your opinion, how guaranteed are the freedoms listed in the Brambell Report for the welfare of captive wild canids, in zoos?

22. Freedom from hunger and thirst

Mark only one oval.

	1	2	3	4	5	
Not guaranteed at all	◯	◯	◯	◯	◯	Totally guaranteed

23. Freedom to have an adequate physical environment

Mark only one oval.

	1	2	3	4	5	
Not guaranteed at all	⬭	⬭	⬭	⬭	⬭	Totally guaranteed

24. Freedom from pain, injury and disease

Mark only one oval.

	1	2	3	4	5	
Not guaranteed at all	⬭	⬭	⬭	⬭	⬭	Totally guaranteed

25. Freedom to express normal behaviours (for the species)

Mark only one oval.

	1	2	3	4	5	
Not guaranteed at all	⬭	⬭	⬭	⬭	⬭	Totally guaranteed

26. Freedom from fear and distress

Mark only one oval.

	1	2	3	4	5	
Not guaranteed at all	⬭	⬭	⬭	⬭	⬭	Totally guaranteed

27. Are you satisfied by the level of welfare of the animals kept in the zoo where you work?

Mark only one oval.

	1	2	3	4	5	
Not satisfied at all	◯	◯	◯	◯	◯	Totally satisfied
